# Regarded as Competent: Medical Knowledge and Professional Acceptance in the Restatement (Third) of Torts

**DOI:** 10.1111/jep.70751

**Published:** 2026-11-12

**Authors:** Boris Nikolic

**Affiliations:** ^1^ Department of Philosophy Heinrich Heine University Düsseldorf Düsseldorf Germany

**Keywords:** clinical practice guidelines, medical custom, medical negligence, philosophy of medicine, respectable minority, Restatement (Third) of Torts

## Abstract

**Rationale, Aims and Objectives:**

The Restatement (Third) of Torts: Medical Malpractice defines the standard of reasonable medical care as ‘the care, skill, and knowledge regarded as competent among similar medical providers’ and lists the state of medical knowledge among the circumstances relevant to it. Its drafters justify a professionally determined standard on epistemic grounds: lay factfinders cannot evaluate medical methods from scratch. This paper asks how, on the Restatement's account, medical knowledge constrains professional judgements of competence when the two conflict.

**Methods:**

Conceptual and doctrinal analysis of the official text of §§ 5 and 6, their comments, illustrations and Reporters' Notes, read against the drafters' stated rationale.

**Results:**

The Restatement expressly incorporates medical knowledge and moves the standard from prevailing practice to professional judgement of competence. It gives little explicit guidance on when professional acceptance that the available evidence does not support ceases to protect a practice. The commentary states its ‘ultimate question’ in terms of what professionals believe would be competent; the respectable‐minority doctrine protects approaches that a respectable group of professionals accepts; and the state of medical knowledge appears among the circumstances of a case rather than as a constraint on the criterion. The respectable‐minority doctrine assigns the plaintiff the burden of persuasion on whether an approach is acceptable, but the commentary does not say what acceptability consists in when authoritative evidence tells against a practice that some providers accept; the guideline provisions of § 6 address compliance but not this converse conflict.

**Conclusions:**

The drafters' epistemic rationale supports a requirement that professional acceptance be justifiable in light of the evidence of benefit and harm, foreseeable risks, available alternatives and the patient's circumstances. Courts deciding whether to adopt § 5, ruling on summary judgment and instructing juries should treat that requirement as part of the standard and determine when evidentially unsupported professional acceptance ceases to protect a practice. This does not require conclusive evidence for every case or the abolition of expert testimony.

## Plain Language Summary

When a patient sues a doctor for negligence, the question is whether the doctor met the ‘standard of care’. For a long time American courts measured this against what doctors usually did. A new statement of the law by the American Law Institute changes the test to what doctors regard as competent, and says that the state of medical knowledge is relevant. This paper asks what happens when the two conflict: when a practice that some doctors accept is not supported, or is contradicted, by the evidence. The new rules do not say clearly which should prevail. The paper argues that the reason courts defer to doctors at all, that doctors know the medical evidence and jurors do not, means that professional acceptance should protect a practice only when it can be justified in light of that evidence, the risks, the alternatives and the patient's situation.

## 1 Introduction

For much of the twentieth century, American courts measured medical negligence against custom: what physicians in good standing ordinarily did. The leading torts treatise described this as a privilege, usually denied to other groups, of setting the profession's own legal standards ‘merely by adopting their own practices’ (Keeton et al. [1], § 32). The custom standard drew criticism for decades [2, 3, 4], and courts gradually moved away from it [5]. The American Law Institute's Restatement (Third) of Torts: Medical Malpractice (hereafter ‘the Restatement’) codifies that movement. Section 5(a) defines the standard of reasonable medical care as ‘the care, skill, and knowledge regarded as competent among similar medical providers in the same or similar circumstances’, and § 5(b)(2) lists ‘the state of medical knowledge and the treatment options available at the time care is provided’ among the circumstances that may be relevant (American Law Institute [ALI] [6], § 5, p. 53). The commentary treats customary practice as evidence of the standard rather than as the standard itself (§ 5 cmts b–c, pp. 54–56).

The Restatement therefore does not ignore medical knowledge. Its Reporters present the state of knowledge as one of two safeguards against the shortcomings of custom (Reporters' Note to § 5 cmt b, p. 68). The question this paper pursues is narrower: how, on the Restatement's account, does medical knowledge constrain professional judgements of competence when the two conflict? Most of the time they do not conflict, because professional judgement usually reflects the evidence. Sometimes they do: a practice continues to be accepted by a respectable group of providers after evidence has accumulated against it, or a practice spreads for reasons that have little to do with its benefits to patients.

I argue that the Restatement leaves this question insufficiently answered. Its commentary states the governing test in terms of what professionals believe would be competent, protects approaches that a respectable minority accepts, and places medical knowledge among the circumstances of a case without saying how it ranks against professional acceptance. The drafters' own rationale for a professionally determined standard is epistemic, and that rationale supports an explicit requirement that professional acceptance be justifiable in light of the evidence and the other considerations competent judgement takes into account. The commentary should state that requirement and say when evidentially unsupported acceptance ceases to protect a practice.

Section 2 sets out what § 5 changed. Section 3 distinguishes three readings of ‘regarded as competent’ and identifies the question the text leaves open. Section 4 examines the two safeguards the Reporters offer. Section 5 turns to proof under § 6, including the respectable‐minority burden structure and the treatment of practice guidelines, and identifies where the commentary needs clarification. Section 6 proposes how the commentary could close it. Section 7 answers objections.

Three limits of scope. First, the Restatement is not binding law. Many jurisdictions still instruct juries in the language of custom; New York's highest court has called customary practice the ‘touchstone’ of malpractice analysis (Nestorowich v. Ricotta [7], as cited in Ref. [6], Reporters' Note to § 5 cmt c, p. 69). Restatements nonetheless shape the development of state law, and a critique of the Restatement's formulations is a critique of a reform proposal with practical consequences. Second, the paper concerns breach, not causation. Third, the analysis uses American doctrine because the Restatement states the standard and its proof in adjacent sections of one document. The question it raises, how a professionally determined standard should relate to the evidence professional judgement is supposed to reflect, arises wherever liability rests on a professional standard.

## 2 What Section 5 Changed

Section 5(a) defines the standard; § 5(b) lists circumstances that may be relevant in a specific case, including the patient's condition, the state of medical knowledge and available treatment options, the resources available to the provider, whether professional standards differ among groups of providers, and any representations the provider made (ALI [6], § 5(b), p. 53). The commentary explains the relationship between the new formulation and the old. Medical professionals usually practise competently, so prevailing practice usually reflects what similar professionals regard as competent; courts and pattern instructions have therefore described the standard as ‘customary’, and in the ordinary case these formulations ‘do not differ in substance’ (§ 5 cmt c, p. 55).

The change matters where prevailing practice and competence come apart. Comment c identifies four such situations: prevailing practice may fall short of what professionals regard as competent; some situations lack an established approach; the content of prevailing practice may be difficult to discern; and particular circumstances, such as innovation or crisis, may justify departure from it. For these cases the comment states the governing test: ‘the ultimate question remains what similar professionals—nationally—believe would be competent to do in the same or similar circumstances’ (§ 5 cmt c, p. 56).

This is a real improvement. A practice that most physicians follow but regard as deficient no longer protects the physician who follows it, and an innovation that peers regard as reasonable no longer exposes its user to liability merely for being unusual (§ 5 cmt g, p. 59). Peters, whose account of the ‘quiet demise’ of deference to custom the Reporters quote, describes the reasonable‐physician standard as one that ‘continues to be physician‐based’ but asks what a reasonable physician would have done rather than what is usually done (Peters [5], as quoted in Ref. [6], Reporters' Note to § 5 cmt c, p. 73). The standard has moved from professional behaviour to professional judgement. What remains to be settled is what makes a professional judgement of competence one the law should accept.

## 3 Three Readings and an Open Question

The phrase ‘regarded as competent among similar medical providers’ can be read in at least three ways.

On an **acceptance reading**, the standard is fixed by what similar providers in fact accept as competent. Acceptance plays the role custom used to play, one step removed from behaviour.

On a **reasonable‐judgement reading**, the standard is what reasonable providers would judge competent, exercising the forms of reasoning the profession recognises. These are not limited to published clinical evidence. Competent judgement also weighs feasibility, available resources, competing risks, the patient's circumstances and preferences, and practical experience, much of which no trial or guideline captures. This is a serious position, and in most cases it is the right description of what the standard asks.

On a **justification‐constrained reading**, the standard is what similar providers could justifiably regard as competent, in light of the evidence of benefit and harm, foreseeable risks, available alternatives and the patient's circumstances. It includes everything the reasonable‐judgement reading includes. It adds one explicit requirement: professional acceptance does not protect a practice that the available evidence shows to expose patients to foreseeable harm that a competent provider, aware of that evidence and of the alternatives, would avoid.

The second and third readings differ only in the conflict case. Where the profession's reasoning has absorbed the evidence, they coincide. They come apart when an accepted practice is contradicted by evidence the profession has been slow to absorb, or when acceptance rests on considerations that do not bear on the patient's interests. The reasonable‐judgement reading leaves the resolution of such cases to the profession's own standards of reasonableness. The justification‐constrained reading requires that acceptance be answerable to reasons a factfinder, with expert help, can assess.

The Restatement's text does not choose between these readings. Several formulations are naturally read as acceptance: the ‘ultimate question’ is what similar professionals ‘believe would be competent’ (§ 5 cmt c, p. 56); under the respectable‐minority doctrine an approach ‘can be regarded as competent and acceptable even if a majority of similar professionals practice or believe differently’ (§ 5 cmt k, p. 61); and the treatise the Reporters quote requires that a school of thought be ‘a recognized one with definite principles (Keeton et al. [1], § 32, as quoted in Ref. [6], Reporters' Note to § 5 cmt k, p. 83). None of these formulations states that acceptance suffices regardless of its justification, and I do not claim the Restatement holds that it does. They are, however, framed in terms of belief and recognition, and none states the constraint that would resolve the conflict case. Section 5(b)(2) makes the state of medical knowledge relevant, but as one circumstance among several, without indicating how it ranks when it tells against what providers accept.

The tourniquet illustrations show where the question arises. A surgeon uses an older tourniquet technique; the plaintiff's expert testifies that it damages nerves and that other techniques are now preferred. Because a respectable minority of competent physicians still uses the older technique, there is no breach. The result changes when the experts testify that the technique is ‘no longer regarded as acceptable’ (§ 5 cmt k, Illustrations 4–5, pp. 61–62). I do not claim the illustrations reach the wrong result; the facts given do not say how strong the evidence of nerve damage is. The illustrations do show that the commentary frames the decisive fact as professional acceptance. They do not address the case in which the evidence of avoidable harm is strong and a respectable minority continues the practice anyway.

The drafters' rationale bears on how that case should be resolved. Comment b explains that medical professionals receive a modified standard ‘not because they have a superior status’ but because a professionally determined standard is an appropriate benchmark where ‘a professional's methods, nearly by definition, will be difficult for lay jurors to evaluate from scratch’ (§ 5 cmt b, p. 55). The Reporters' Note gives the conventional formulation: the informed approach of professionals to matters outside common knowledge should not be ‘evaluated by the ad hoc judgments of a lay judge or lay jurors aided by hindsight’ (Osborn v. Irwin Memorial Blood Bank [8], as quoted in Ref. [6], Reporters' Note to § 5 cmt b, p. 67). This is an epistemic justification: it supports deference to professional judgement because, and in so far as, that judgement reflects knowledge and reasoning jurors lack. It does not support deference to acceptance that the available evidence shows to be mistaken. The rationale therefore supports the justification‐constrained reading, and it gives the drafters a reason to state the constraint rather than leave it implicit.

## 4 The Reporters' Safeguards

The drafters were aware that professional standards can drift. The Reporters' Note to comment b sets out the concern that custom‐based standards may shortchange patients' interests, including Henderson's argument that defensive medicine and custom‐based legal standards can form a ‘feedback loop’: defensive practices become customary, the custom raises the legal standard, and the raised standard encourages further defensive practice (Henderson [9], as discussed in Ref. [6], Reporters' Note to § 5 cmt b, p. 68). The Note states that the Section guards against such concerns in two ways: what similar professionals regard as competent, ‘rather than simply their raw behavior’, ultimately determines the standard; and § 5(b)(2) makes the state of medical knowledge a factor in determining when care is incompetent (p. 68).

Both safeguards point in the right direction. Neither settles the conflict case. The first corrects practice towards professional judgement. It helps where physicians follow a practice they themselves regard as deficient, a gap the empirical literature documents [10]. It helps less where judgement itself has drifted, and the feedback loop the Reporters describe is one way it can: a defensive practice that becomes widespread can come to be regarded as what a careful physician does. The second makes the state of knowledge relevant and indexes the standard to the time of care, which protects providers against hindsight. As draughted, it does not say what follows when the state of knowledge tells against an accepted practice.

## 5 Proof: Where the Gap Lies

Section 6(a) provides that breach ‘must be established through the testimony of a qualified expert’, except where res ipsa loquitur applies, where breach is a matter of common knowledge, or where the provider admits breach (ALI [6], § 6(a), p. 89). Under comment o to § 5, the judge decides whether expert testimony is required and frames the enquiry, and the factfinder resolves disputed facts and ‘any disagreement among experts’ about whether the standard was met (§ 5 cmt o, p. 65).

I do not argue that expert testimony is unreliable, that juries cannot evaluate it, or that one expert is better than two. Adversarial testing of qualified experts after admissibility screening and summary judgement can be a good way to resolve reasonable disagreement. The question is what the factfinder is told the disagreement is about. The respectable‐minority doctrine supplies a burden structure: the defendant bears the burden of identifying a second school of thought and producing sufficient evidence of its existence; the plaintiff bears the burden of persuasion on whether the defendant's approach is acceptable (§ 5 cmt k, p. 61). That allocation leaves room for the enquiry this paper proposes. A factfinder asked whether an approach is acceptable could find that it is not because its acceptance is not justified by the evidence. The difficulty is that the commentary does not say so, and the surrounding formulations point the other way: the doctrine is introduced as protecting an approach that can be ‘regarded as competent and acceptable’ by a respectable group, and in the tourniquet illustrations the decisive testimony is that the technique is or is not ‘regarded as acceptable’ (§ 5 cmt k and Illustrations 4–5, pp. 61–62). Suppose the defence expert testifies that a respectable minority accepts the defendant's approach, and the plaintiff's expert, relying on authoritative evidence, testifies that the approach exposes patients to avoidable harm. Nothing in the commentary prevents the factfinder from preferring the plaintiff's account; nothing tells it whether the question is the existence of professional acceptance or its justification. The gap is one of insufficiently explicit guidance, not a doctrinal barrier, and it falls exactly where the guidance is needed.

The guideline provisions show the same gap from the other side. Section 6(b) provides that evidence that the provider complied with a relevant and authoritative practice guideline may be used to rebut the plaintiff's claim of breach, or may be sufficient to find that the provider met an acceptable alternative standard of care under § 5 comment k (§ 6(b), p. 89). Comment f describes the provision as ‘asymmetric’: guidelines may reinforce or rebut a plaintiff's expert, but the Subsection ‘does not permit an authoritative practice guideline, by itself, to establish a violation of the standard of care’ (§ 6 cmt f, p. 95). Its reasons are that guidelines often recommend more than competence requires, and that where respectable views differ a guideline may capture only one of several acceptable approaches (p. 95).

These provisions do not prevent a plaintiff from relying on a guideline. A plaintiff whose expert relies on an authoritative guideline can reach the factfinder now, and the reasons comment f gives for the asymmetry are sound reasons why departure from a guideline is not, by itself, breach. The point is different. Section 6(b)(2) allows compliance with an authoritative guideline to establish an acceptable alternative standard: the guideline functions as evidence of a school of thought. The commentary does not address the converse question, whether an authoritative guideline and the evidence behind it can show that an approach accepted by a respectable minority is not acceptable. Comment f's reasons do not answer it. A guideline recommending more than competence requires does not show that a practice it advises against is competent; and that respectable views differ does not show that every view a respectable group holds is justifiable.

Two observations in the Reporters' own account bear on that question. One reason they give for permitting defensive reliance on guidelines is that aggregated expert views ‘may help avoid the subjective elements that can characterize an individual and hand‐picked litigation expert's judgments’ (§ 6 cmt f, p. 94). And they note that medical defendants generally face less difficulty than plaintiffs in finding experts (Reporters' Note to § 6 cmt f, p. 101). The epistemic value of aggregated evidence does not depend on which party offers it. It is therefore relevant to the conflict question in the same way it is relevant to compliance, even if the asymmetry on sufficiency is retained.

The Restatement also builds one illustration on facts close to *Helling v. Carey* [11], the Washington decision that found negligence despite unanimous professional practice in a glaucoma‐screening case. In Illustration 2 the plaintiff, without expert testimony, relies on a common‐sense cost‐benefit argument and loses (§ 6 cmt b, pp. 90–91); the Reporters' Note observes that the illustration uses similar facts but reaches a different result (p. 96). The justification‐constrained reading does not revive *Helling*. *Helling* substituted the court's own cost‐benefit judgement for professional judgement. The constraint proposed here operates through expert evidence about what the clinical evidence and the other relevant considerations support.

## 6 What the Commentary Should Specify

The gap could be closed by clarification, without changing the black letter. The commentary to § 5 could state that professional acceptance of an approach does not establish that it meets the standard of care where the available evidence shows that the approach exposes patients to foreseeable harm that a competent provider, aware of that evidence and of the available alternatives, would avoid. Comment c's ‘ultimate question’ would then ask what similar professionals could justifiably regard as competent; comment k would make clear that the acceptability on which the plaintiff bears the burden of persuasion concerns whether the approach is justifiable, not only whether a respectable group accepts it; and comment f would note that authoritative guidelines, with expert testimony, may be used to show that an approach accepted by some providers does not meet that condition. Several clarifications follow.

First, the constraint is not a requirement of published evidence for every choice. Competent judgement weighs the strength and direction of the evidence of benefit and harm, the magnitude of foreseeable risks, the alternatives available, the patient's condition, circumstances and preferences, and the resources at hand. Clinical experience and feasibility are legitimate considerations. The constraint applies to the conflict case: it identifies when acceptance stops protecting a practice, not what every competent choice must rest on.

Second, uncertainty does not automatically widen the range of competent choices. Where evidence of benefit is weak and the risks are substantial, restraint may be the only justifiable course. Where evidence is absent, the justification of a choice shifts to reasoning about foreseeable harms, alternatives and the patient's situation; the absence of research does not make every professionally endorsed choice defensible. Where evidence is strong and consistent, it leaves little room for divergent practice.

Third, custom and professional acceptance keep an evidential role. Prevailing practice and peer acceptance are often good evidence that an approach is justifiable, because they usually reflect experience and the literature. They cease to be decisive only where the evidence shows they have gone wrong.

Fourth, expert testimony and adversarial testing remain. Experts are needed to identify, interpret and apply the evidence and the other considerations. What the commentary would change is the question the factfinder is told to resolve in the conflict case: whether the defendant's approach is justifiable, not merely whether a respectable group accepts it. How evidence is best put before the factfinder, whether through party experts, court‐appointed experts under Federal Rule of Evidence 706 [12] or other institutional means, is a separate question this paper does not resolve.

This position should be distinguished from two others. Williams argued in 2004 that the legal standard of care would and should absorb the concepts of evidence‐based medicine, and Massie defended the professional standard in reply [13, 14]. That debate concerned whether the law should adopt an evidence‐based standard in place of the professional one. The argument here accepts the professional standard and asks only that the Restatement make explicit a constraint its own rationale supports. It also differs from Peters's assessment of the reform as a real improvement [15]. I agree that it is one; the disagreement concerns whether it is sufficiently specified for the cases in which professional acceptance and medical knowledge conflict.

## 7 Objections

### 7.1 The Constraint Is Already Implicit

One might reply that the reasonable‐judgement reading, and the plaintiff's burden on acceptability, already include it. If so, the clarification changes the law little, and its benefit is guidance: courts, counsel and experts would know that in the conflict case the question is whether acceptance is justified, and the commentary would match the rationale the drafters give for it. The clarification also has possible costs. It could invite hindsight, by encouraging the factfinder to judge past care by evidence that became clear only later, and it could be read as licensing closer judicial scrutiny of clinical judgement. Both risks are limited by the terms of the proposal: § 5(b)(2) indexes the enquiry to the evidence available at the time of care; the constraint concerns foreseeable harm; the plaintiff retains the burden of persuasion; and the allocation of functions between judge and factfinder is unchanged. Without the clarification, the formulations a court is most likely to quote, those of comments c and k, are framed in the language of belief, acceptance and recognition, and a constraint implicit only in the rationale is less likely to guide the conflict case.

### 7.2 Overtreatment

The asymmetric treatment of guidelines is meant in part to counter the defensive‐medicine feedback loop (Reporters' Note to § 6 cmt f, pp. 102–103). The constraint supports that aim. Where the evidence supports restraint, a justification‐constrained standard protects the less intensive choice whatever prevailing practice may be, and it does so whether or not an authoritative guideline exists. It also applies against defensive practices that have come to be accepted without evidence of benefit.

### 7.3 Institutional Competence

The institutional objection is that lay factfinders should not substitute unaided clinical judgements for the reasoned judgements of qualified professionals (cf. Restatement (Second) of Torts § 299A cmt f [16], as quoted in Ref. [6], § 5 cmt k, p. 61). The constraint does not ask them to. The allocation of functions in comment o is unchanged, and the factfinder already resolves disagreement about acceptability under comment k's burden structure. The requirement that acceptance be justifiable applies whether or not the evidence is contested. Where it is contested, the factfinder decides, on the expert testimony, whether the plaintiff has shown that the defendant's approach was not justifiable; reasonable disagreement about the evidence can support a finding that the care was competent. Disagreement bears on whether the plaintiff has carried the burden, not on whether the requirement applies.

### 7.4 Fallible Evidence

Published evidence can be biased, incomplete or unrepresentative of the patients whose care is later judged by it [17, 18], and the Reporters note that guidelines may reflect professional or industry interests (Reporters' Note to § 6 cmt f, p. 104). The constraint requires that the evidence be assessed, and weak or compromised evidence will not show avoidable harm. It does not follow that acceptance resting on no better grounding should prevail.

### 7.5 Not the Law

Many jurisdictions still apply custom‐based instructions. Restatements, however, are written to guide courts, and the formulations the commentary uses will shape how the reform is received. Specifying the constraint now is more useful than correcting the formulations after courts have adopted them.

## 8 Conclusion

The Restatement (Third) of Torts: Medical Malpractice moves the standard of reasonable medical care from what physicians do to what they judge to be competent, and it makes the state of medical knowledge relevant. It gives little explicit guidance on how medical knowledge constrains professional judgement when the two conflict, and its commentary frames the decisive questions in the language of belief, acceptance and recognition. The drafters justify a professionally determined standard because professionals know what lay factfinders cannot. That justification supports an explicit requirement that professional acceptance be justifiable in light of the evidence, foreseeable risks, alternatives and the patient's circumstances. Courts deciding whether to adopt § 5, ruling on summary judgment and instructing juries should treat that requirement as part of the standard and determine when evidentially unsupported professional acceptance ceases to protect a practice.

## Ethics Statement

Ethics approval was not required because this manuscript is a conceptual and normative analysis and did not involve human participants, animals, identifiable personal data, or primary data collection.

## Conflicts of Interest

The author declares no conflicts of interest.

## Use of AI

Generative AI (Anthropic's Claude) was used for language editing and formatting.

## Data Availability

Data sharing is not applicable to this article because no datasets were generated or analysed during the current study.
